# Leadless Versus Transvenous Single‐Chamber Ventricular Pacemakers: Real‐World Evidence From Aveir VR Coverage With Evidence Development Study

**DOI:** 10.1161/JAHA.125.042471

**Published:** 2025-10-23

**Authors:** James E. Ip, Peter A. Brady, Anne M. Kroman, Blandine Mondésert, Leonard Ganz, Jennifer M. Joseph, Deepti Bettampadi, Yajing Hu, Yelena Nabutovsky, Rahul N. Doshi

**Affiliations:** ^1^ Weill Cornell Medicine New York Presbyterian Hospital Division of Cardiology New York NY; ^2^ Advocate Health Chicago IL; ^3^ Medical University of South Carolina Charleston SC; ^4^ Montreal Heart Institute Université de Montréal Montreal Quebec Canada; ^5^ Abbott Sylmar CA; ^6^ Abbott Santa Clara CA; ^7^ HonorHealth Scottsdale AZ; ^8^ University of Arizona College of Medicine Phoenix Phoenix AZ

**Keywords:** Aveir, leadless pacemakers, outcomes, reinterventions, safety, transvenous pacemakers, Electrophysiology

## Abstract

**Background:**

Aveir VR, a helix‐fixation leadless pacemaker (LP), was US Food and Drug Administration (FDA)‐approved in 2022. The performance of this novel LP has not been evaluated against transvenous single chamber ventricular pacemakers (TV‐VVI) in a real‐world setting. This observational coverage with evidence development study of Aveir VR implants (Aveir coverage with evidence development) evaluates complications and outcomes among Medicare beneficiaries implanted with Aveir VR immediately after FDA approval compared with those implanted with TV‐VVI.

**Methods:**

Medicare fee‐for‐service and Abbott device registration data were used to identify de novo Aveir VR LP (implanted 6/2022–6/2024) or TV‐VVI (all manufacturers, implanted 1/2021–6/2024). Outcomes evaluated included 30‐day and 12‐month complications, reinterventions, and mortality rates, adjusted for demographics, comorbidities, and hospital encounter characteristics.

**Results:**

Among 2425 Aveir VR LP and 21 335 TV‐VVI patients included in this analysis, Aveir VR patients had significantly fewer acute device‐related complications (1.4% versus 2.8%, *P*=0.002). Overall acute complications were similar between Aveir VR (6.5%) and TV‐VVI (6.8%), *P*=0.69. Cardiac effusion/perforation rates were also similar between Aveir VR (0.4%) and TV‐VVI (0.3%), *P*=0.45. Aveir VR had a 30% lower overall 12‐month complication rate compared with TV‐VVI (4.1% versus 5.7%, *P*=0.02), including a 47% lower device‐related complication rate (2.8% versus 5.2%, *P*=0.0002). There were 61% fewer 12‐month reinterventions with Aveir VR compared with TV‐VVI (1.2% versus 3.2%, *P*<0.0001). Finally, Aveir maintained a comparable 30‐day mortality but a lower 12‐month mortality rate (17.0% versus 18.2%, *P*=0.02).

**Conclusions:**

This real‐world evidence study, conducted immediately after commercialization of Aveir VR, demonstrates that safety of helix‐fixation LPs is better than traditional TV‐VVI pacemakers.

**Nonstandard Abbreviations and Acronyms**
CED: coverage with evidence development
CMS: The Centers for Medicare & Medicaid Services
FDA: US Food and Drug Administration
FFS: fee‐for‐service
LP: leadless cardiac pacemaker
TV‐VVI: transvenous single chamber ventricular pacemakers

**Clinical Perspectives**

**What Is New?**

- This observational coverage with evidence development study of single chamber, helix‐fixation ventricular leadless pacemakers (Aveir coverage with evidence development) evaluated the complications and outcomes among Medicare beneficiaries implanted with Aveir VR immediately after US Food and Drug Administration approval in 2022 compared with those implanted with transvenous single chamber ventricular pacemakers.
- Compared with transvenous systems, Aveir VR was associated with lower rates of acute device‐related complications, as well as reduced overall complications, device‐related events, reinterventions, and mortality at 12 months.

**What Are the Clinical Implications?**

- This real‐world evidence study, conducted immediately after commercialization, highlights the improved outcomes of helix‐fixation, Aveir VR leadless pacemakers compared with traditional transvenous pacemakers for patients with pacing needs.

As leadless cardiac pacemaker (LP) technology advances with the development of novel devices and as physicians learn new procedural techniques, it is imperative to continually assess the safety and reliability of each iteration. The first LPs developed were single chamber ventricular devices that utilized different fixation mechanisms: one involving a helix and another involving tines; both were found to be safe and effective.^1^, ^2^ The Aveir ventricular (Aveir VR) LP, which uses the helix mechanism, received US Food and Drug Administration (FDA) approval in June 2022 following demonstration of its safety and efficacy in the Leadless II trial.^1^ This system also provides the foundation for the more recently approved dual chamber LP system (Aveir DR).^3^

Centers for Medicare & Medicaid Services (CMS), as part of the national coverage determination, may assign coverage of an item or service only in the context of a clinical study. LPs are included under this national coverage determination, which mandates a coverage with evidence development (CED) study to address evidence gaps identified by CMS. Specifically, the national coverage determination requires a comparison of LP versus transvenous pacemaker complication rates and patient outcomes. Ongoing LP CEDs include the Micra CED (Medtronic), in which the first report showed that leadless pacemakers with tine fixation had higher rates of acute pericardial effusion or perforation and a lower rate of 6‐month complications compared with transvenous pacemakers.^4^ The 3‐year follow‐up report also found lower chronic complications as well as reintervention rates for LPs compared with transvenous pacemakers.^5^ The present study, the Aveir VR leadless pacemaker CED study, continually evaluates complications and outcomes among Medicare beneficiaries implanted with Aveir VR compared with those implanted with a transvenous single chamber ventricular pacemaker. The CED study protocol was reviewed and approved by CMS (June 21, 2022), and the study is required to continue until CMS provides final coverage determination.

Due to the design and implant procedure differences between Micra and Aveir LPs,^6^ it is essential to evaluate the real‐world use of this technology post‐commercialization. This observational Aveir VR CED study compares the 30‐day and 12‐month complication, reintervention, and mortality rates between the Aveir VR and single‐chamber transvenous pacemakers (TV‐VVI) soon after FDA approval of Aveir VR.

## METHODS

### Study Design and Data Sources

Data for this study were used under a data use agreement and therefore cannot be shared. Centers for Medicare and Medicaid Services data can be requested under an approved research protocol via ResDAC (www.resdac.org). The Aveir VR CED study is a comparative observational study using the most currently available Medicare fee‐for‐service (FFS) insurance claims and Abbott device registration data. Medicare data files included institutional claims (inpatient, outpatient), non‐institutional claims (carrier), and Master Beneficiary Summary Files, which contain demographics, birth and death dates, Medicare eligibility status, and enrollment dates. Abbott device registration data contained date of birth, sex, device type, implant dates, and implanting facility.

### Study Population

The study included 2 groups of patients implanted with a single chamber pacemaker: 1. Aveir VR LP (models LSP112V and LSP202V; Abbott) implanted after CMS approval of the Aveir VR CED study (June 21, 2022, to June 30, 2024); and 2. TV‐VVI implants from all manufacturers as the control group (January 1, 2021, to June 30, 2024).

Patients implanted with an LP or TV‐VVI in the inpatient setting were identified in Medicare inpatient claims using *International Classification of Diseases, 10th Revision, Procedure Coding System (ICD‐10‐PCS)* (Table S1), and patients implanted in the outpatient setting were identified in Medicare outpatient claims using Current Procedural Terminology (CPT) Fourth Edition, Healthcare Common Procedure Coding System (Table S1). Of patients implanted with an LP, those implanted with Aveir VR LP were then identified by linking Medicare with Abbott device registration data using probabilistic linking.^7^ Linking variables included date of birth, sex, device type, implant dates, and implanting facility. The index date for both study groups was the implant procedure date from Medicare claims.

Inclusion criteria required patients to have at least 12 months of continuous Medicare FFS coverage before the implant, and coverage for at least 30 days post‐implant or until death if within 30 days. The 12‐month period before the implant allows for collection of clinical characteristics from the claims data, such as comorbidities and history of relevant procedures. Implants were also required to be de novo; patients who had other cardiac implantable electronic device implants (Table S2) before the index date were excluded from the study.

This study was conducted as a retrospective analysis of de‐identified data. The study was granted a full waiver of informed consent and a HIPAA waiver from Western IRB and is registered on ClinicalTrials.gov (NCT05336877).^8^

### Outcome Measures

Primary outcome measures included (1) overall acute complication rate, defined as peri‐procedural adverse events occurring within 30 days of implant and (2) all‐cause mortality rate within 30 days of implant. The following acute complications were included in overall acute complication rate: deep vein thrombosis (DVT), pulmonary embolism, thrombosis, embolism, arteriovenous fistula, vascular pseudoaneurysm, pericardial effusion, cardiac perforation, cardiac tamponade, device dislodgement or displacement, infection, hemorrhage, device malfunction, pain, stenosis, pocket complication, postprocedural hematoma, postprocedural hemorrhage, pericarditis, acute myocardial infarction, intraoperative cardiac arrest, bleeding or failure of vascular closure device requiring intervention, hemothorax, and pneumothorax. Complications were identified by the presence of a diagnosis code indicative of a device‐ or procedure‐related complication. Diagnosis codes could appear in any position on the claim: as the primary diagnosis or any of the secondary diagnoses. Post‐implant infections and DVTs were considered complications only if the patient did not have these diagnoses in the 30 days before their index implant. See Table S3 for individual acute complication diagnosis codes and operational definitions.

Secondary outcome measures included (1) overall chronic complication rate, defined as post‐procedural adverse events occurring within 12 months of implant, (2) device‐related reintervention rate occurring within 12 months of implant and (3) all‐cause mortality rate occurring within 12 months of implant. The following chronic complications were included in the overall chronic complication rate: thrombosis, embolism, device malfunction, device dislodgement or displacement, infection, hemorrhage, pain, stenosis, pocket complication, pericarditis, and hemothorax. Chronic complications were identified by the presence of a diagnosis code that indicates a device‐ or procedure‐related adverse event. Diagnosis codes could appear in any position on the claim: as the primary diagnosis or as any of the secondary diagnoses. Device‐related reinterventions were identified by presence of a procedure code indicating a new pacemaker implant or device replacement with a single chamber ventricular transvenous or leadless device, device revision, or device explant. See Table S4 for individual chronic complication diagnosis codes and operational definitions. See Table S5 for device‐related reintervention procedure codes and operational definitions.

### Statistical Analysis

Baseline characteristics obtained from Medicare FFS claims in the 12 months before index implant included demographics (age, sex, race/ethnicity, dual eligibility status for Medicare and Medicaid), clinical characteristics (see Table S6 for diagnosis and procedure codes), and health care encounter characteristics (number of days from admission to implant and indicator variables for inpatient implant, admission through emergency room, temporary pacing during hospitalization, and weekend implant). Differences in these baseline characteristics were compared between Aveir VR LP and TV‐VVI patients using *t*‐tests for continuous variables and *χ* ^2^ tests for categorical variables.

Propensity score overlap weights were used to adjust for differences in baseline characteristics between Aveir VR LP and TV‐VVI patients. The propensity scores were constructed by fitting a logistic regression model where the binary outcome was whether or not the patient was implanted with an Aveir VR, while the predictors were demographic and clinical characteristics listed in Table 1. Goodness‐of‐fit tests were evaluated to ensure the model was correctly specified. Overlap weights were then calculated from the propensity scores based on methods described by Li et al, which emphasizes patients with the most overlap in their observed characteristics (see Figure S1 for diagnostics, such as the standardized difference before and after weighting and a graph of the propensity scores).^9^ The acute complication rate was compared between Aveir VR LP and TV‐VVI patients with unadjusted and adjusted logistic regression models. The chronic complication rate and device reintervention rate were compared between the study groups with unadjusted and adjusted Fine‐Gray competing risk models, with death as the competing risk. The 30‐day and 12‐month mortality rates were compared between the study groups with unadjusted and adjusted Cox proportional hazard models. All adjusted models included the treatment variable (Aveir VR LP or TV‐VVI) as the predictor, and the normalized propensity score overlap weight was included in the weight statement. No additional covariates were included as predictors in the models. For all Fine‐Gray competing risk and Cox proportional hazard models, patients were censored at the earliest of the following events: (1) after experiencing the outcome, (2) 12 months after implant, (3) end of claims data availability, (4) end of Medicare FFS enrollment, (5) cardiac implantable electronic device implant, including leadless single‐chamber pacemaker, transvenous single‐chamber pacemaker, dual‐chamber leadless pacemaker, dual‐chamber transvenous pacemaker, implantable cardioverter defibrillator (ICD), cardiac resynchronization therapy pacemaker (CRT‐P), or defibrillator (CRT‐D), (6) leadless device explant, transvenous pacemaker system explant or battery removal/replacement, (7) death. For all outcomes, events were counted towards the event rates before the patient was censored. All analyses were conducted in SAS Enterprise Guide version 7.15 (SAS Institute Inc).

**Table 1:** Baseline Characteristics

	Aveir VR	Transvenous	*P* value
	(N=2425) n (%)	(N=21 335) n (%)	
Demographic characteristics			
Age, mean±SD, y	80.0±8.8	82.9±7.7	<0.01
Female sex	991 (40.9%)	9078 (42.5%)	0.11
Race or ethnicity			<0.01
White	2151 (88.7%)	19 740 (92.5%)	
Black	124 (5.1%)	733 (3.4%)	
Asian/Hispanic/Native American	81 (3.3%)	428 (2.0%)	
Other/Unknown	69 (2.8%)	434 (2.0%)	
Hospital encounter characteristics			
Inpatient implant	1133 (46.7%)	10 819 (50.7%)	<0.01
Admission to implant, mean±SD, d	2.0±4.8	1.6±3.2	<0.01
Weekend implant	24 (1.0%)	590 (2.8%)	<0.01
Admission through emergency room	680 (28.0%)	7291 (34.2%)	<0.01
Temporary pacing during hospitalization	124 (5.1%)	1151 (5.4%)	0.56
Clinical characteristics			
Atrial and ventricular arrhythmias			
Atrial fibrillation	2014 (83.1%)	20 257 (94.9%)	<0.01
Atrial flutter	624 (25.7%)	4967 (23.3%)	<0.01
Ventricular arrhythmia	492 (20.3%)	4602 (21.6%)	0.15
Atrioventricular block	1175 (48.5%)	9060 (42.5%)	<0.01
Sinus node dysfunction	1543 (63.6%)	13 408 (62.8%)	0.45
Charlson Comorbidity Index, mean±SD	5.0±3.4	4.6±3.1	<0.01
Chronic obstructive pulmonary disease	667 (27.5%)	5939 (27.8%)	0.73
Coronary artery disease	1395 (57.5%)	12 115 (56.8%)	0.48
Diabetes	1065 (43.9%)	8778 (41.1%)	<0.01
Heart failure	1309 (54.0%)	13 323 (62.4%)	<0.01
Hyperlipidemia	2053 (84.7%)	17 802 (83.4%)	0.12
Hypertension	2287 (94.3%)	20 564 (96.4%)	<0.01
Peripheral vascular disease	683 (28.2%)	5857 (27.5%)	0.46
Recent infection due to cardiac implants/prosthetic devices	<11*	18 (0.1%)	<0.01
COVID‐19 within 30 d before implant	62 (2.6%)	702 (3.3%)	0.05
Prior cardiovascular events and procedures			
Prior coronary artery bypass graft	292 (12.0%)	2806 (13.2%)	0.12
Prior acute myocardial infarction	499 (20.6%)	4092 (19.2%)	0.1
Prior percutaneous coronary intervention	374 (15.4%)	3285 (15.4%)	0.97
Concomitant atrial ablation	329 (13.6%)	2579 (12.1%)	0.04
Concomitant TAVR	52 (2.1%)	576 (2.7%)	0.11
Prior TAVR	83 (3.4%)	888 (4.2%)	0.08
Renal disease	1272 (52.5%)	11 610 (54.4%)	0.07
End‐stage renal disease	202 (8.3%)	797 (3.7%)	<0.01
Dialysis dependence	170 (7.0%)	498 (2.3%)	<0.01
Tricuspid valve disease	882 (36.4%)	8728 (40.9%)	<0.01
Tricuspid valve regurgitation	565 (23.3%)	5211 (24.4%)	0.22

TAVR, transcatheter aortic valve replacement.

* To comply with the Centers for Medicare and Medicaid Services cell size suppression policy, a cell containing a value of 1 to 10 cannot be reported directly; therefore, “<11” is used to display a value of 1 to 10.

## RESULTS

### Patient Characteristics

**Figure 1:**
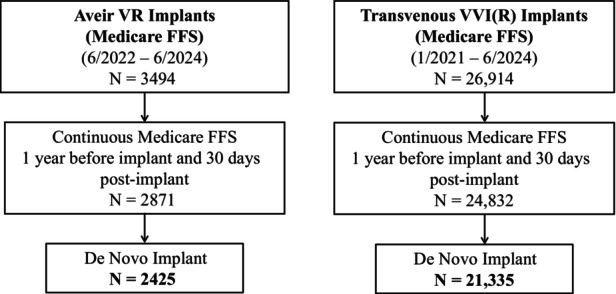
Cohort diagram. Patients were included in the study if they were Medicare beneficiaries implanted with a leadless pacemaker, Aveir ventricular (Aveir VR), or a transvenous single chamber ventricular pacemaker (transvenous VVI). Inclusion criteria required patients to have at least 12 months of continuous Medicare FFS coverage before the implant, and coverage for at least 30 days post‐implant or until death if within 30 days. Implants were required to be de novo; patients who had other cardiac implantable electronic device implants before the index date were excluded from the study. FFS, fee‐for‐service.

A total of 30 408 Medicare FFS beneficiaries were identified (Aveir VR LP, n=3494; TV‐VVI, n=26 914). After excluding patients who did not have at least 12 months of continuous Medicare FFS coverage before implant and 30 days post‐implant or had a prior cardiac implantable electronic device, there were 2425 Aveir VR LP and 21 335 TV‐VVI patients included in this analysis (Figure 1). Mean (min, max) follow‐up was 228 (0, 365) days for Aveir VR LP and 272 (0, 365) days for TV‐VVI. Differences in baseline characteristics were observed between the 2 study groups, as shown in Table 1. Compared with TV‐VVI, Aveir VR LP patients were slightly younger (80.0±8.8 years versus 82.9±7.7 years, *P*<0.01) and more racially diverse. In the Aveir VR group, there were statistically fewer inpatient and weekend implants compared with TV‐VVI, but they still comprised nearly half of all Aveir VR implants.

Regarding the clinical characteristics of single chamber pacemaker patients, Aveir VR LP patients had less heart failure (54% versus 62.4%, *P*<0.01) and tricuspid valve disease (36.4% versus 40.9%, *P*<0.01), but more diabetes (43.9% versus 41.1%, *P*<0.01), end‐stage renal disease (8.3% versus 3.7%, *P*<0.01), and dialysis dependence (7.0% versus 2.3%, *P*<0.01). The higher comorbidities were reflected by the higher Charlson comorbidity index among Aveir VR patients (5.0±3.4 versus 4.6±3.1, *P*<0.01).

### Acute Complications

Overall and individual acute complication rates are shown in Table 2. Before and after adjustment, there was no significant difference in overall acute complications between Aveir VR LP and TV‐VVI (adjusted rate 6.5% versus 6.8%; odds ratio, 0.95 [95% CI, 0.86–1.05]). Aveir VR LP had significantly lower acute device‐related complications (adjusted rate 1.4% versus 2.8%, *P=*0.002; adjusted OR, 0.49 [95% CI, 0.40–0.59]), which is a 51% risk reduction compared with TV‐VVI. This is in part because of a lower rate of hemorrhage for Aveir VR LP (adjusted rate 0.04 versus 0.4, *P=*0.01). There was a 0.6% rate of dislodgment with Aveir VR, similar to the 0.8% rate observed with TV‐VVI (*P=*0.55). Cardiac effusion/perforation rates were similar in both groups (0.4% with Aveir VR versus 0.3% with TV‐VVI, *P=*0.45). There was a higher rate of DVT in the Aveir VR LP group, which was not significant after adjustment (1.6% with Aveir VR versus 1.0% with TV‐VVI, *P=*0.08).

**Table 2:** Unadjusted and Adjusted 30‐Day Complication Rates

	Unadjusted, n (%)			Adjusted, %		
	Aveir VR	Transvenous	*P* value	Aveir VR	Transvenous	*P* value
	(N=2425)	(N=21 335)		(N=2425)	(N=21 335)	
Overall complications	6.7%	6.3%	0.45	6.5%	6.8%	0.69
Embolism and thrombosis	69 (2.9)	429 (2.0)	0.007	2.8	2.1	0.31
Deep vein thrombosis	40 (1.7)	186 (0.9)	0.0002	1.6	1	0.08
Pulmonary embolism	33 (1.4)	254 (1.2)	0.47	1.4	1.3	0.97
Thrombosis due to cardiac device	<11*	<11*	0.83	0.03	0.03	0.97
Embolism due to cardiac device	<11*	<11*	0.001	0.09	0.003	0.21
Events at puncture site	29 (1.2)	67 (0.3)	<0.0001	1.2	0.4	0.008
Cardiac effusion/perforation	<11*	57 (0.3)	0.2	0.4	0.3	0.45
Device‐related complication	34 (1.4)	546 (2.6)	0.001	1.4	2.8	0.002
Device dislodgement	15 (0.6)	151 (0.7)	0.62	0.6	0.8	0.55
Infection/inflammatory reaction	<11*	74 (0.4)	0.25	0.2	0.5	0.08
Hemorrhage due to cardiac device	<11*	101 (0.5)	0.002	0.04	0.4	0.01
Device malfunction	16 (0.7)	183 (0.9)	0.31	0.7	1	0.28
Pain due to cardiac device	0	26 (0.1)	0.09	0	0.1	0.11
Stenosis due to cardiac device	<11*	33 (0.2)	0.16	0.04	0.2	0.24
Pocket complication	N/A	107 (0.5)	…	N/A	…	…
Other complications	36 (1.5)	328 (1.5)	0.84	1.4	1.5	0.76
Postprocedural hematoma	<11*	67 (0.3)	0.2	0.2	0.3	0.5
Postprocedural hemorrhage	<11*	15 (0.1)	<0.0001	0.3	0.1	0.17
Pericarditis	20 (0.8)	46 (0.2)	<0.0001	0.8	0.2	0.01
Acute myocardial infarction	<11*	15 (0.1)	0.37	0.1	0.1	0.72
Intraoperative cardiac arrest	<11*	26 (0.1)	0.59	0.1	0.1	0.8
Bleeding or failure of vascular closure device requiring intervention	<11*	20 (0.1)	0.0002	0.3	0.1	0.18
Hemothorax	0	<11 (<0.05%)	…	0	…	…
Pneumothorax	…	160 (0.7)	…	…	…	…

* To comply with the Centers for Medicare and Medicaid Services cell size suppression policy, a cell containing a value of 1 to 10 cannot be reported directly; therefore, “<11” is used to display a value of 1 to 10.

### Chronic Complications

**Figure 2:**
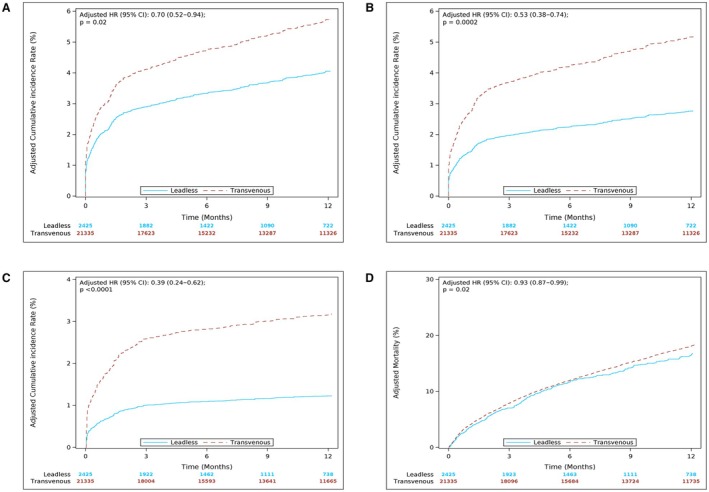
Time‐to‐event graphs of 12‐month complications, device‐related complications, device reinterventions, mortality. (**A**) **12‐month complication rates**. HRs and 95% CIs were based on adjusted Fine‐Gray competing risk models. Patients with an Aveir ventricular (Aveir VR) pacemaker (leadless) had a significantly lower rate compared with patients with a transvenous pacemaker. (**B**) **12‐month device‐related complication rates.** HRs and 95% CIs were based on adjusted Fine‐Gray competing risk models. Patients with an Aveir ventricular (Aveir VR) pacemaker (leadless) had a significantly lower rate compared with patients with a transvenous pacemaker. (**C**) **12‐month device reintervention rates.** HRs and 95% CIs were based on adjusted Fine‐Gray competing risk models. Patients with an Aveir ventricular (Aveir VR) pacemaker (leadless) had a significantly lower rate compared with patients with a transvenous pacemaker. (**D**) **12‐month all‐cause mortality rates**. HRs and 95% CIs were based on adjusted Cox proportional hazard models. Patients with an Aveir ventricular (Aveir VR) pacemaker (leadless) had a significantly lower rate compared with patients with a transvenous pacemaker. HR, hazard ratio.

Overall (Figure 2A) and individual chronic complication rates at 12 months are shown in Table 3. Compared with TV‐VVI, Aveir VR’s overall chronic complication rate trended lower before adjustment. After adjustment, there was a significant 30% lower risk of overall chronic complications for Aveir VR LP compared with TV‐VVI (adjusted rate 4.1% versus 5.7%; hazard ratio, 0.70 [95% CI, 0.52–0.94]). Aveir VR had a 47% lower risk of device‐related complications at 12 months compared with transvenous pacemakers (adjusted rate 2.8% versus 5.2%; adjusted hazard ratio, 0.53 [95% CI, 0.38–0.74]) (Figure 2B). This was in part because of a lower rate of infection (adjusted rate 0.4% versus 1.4%, *P=*0.002), hemorrhage (adjusted rate 0.05% versus 0.5%, *P=*0.03), and pain (adjusted rate 0% versus 0.2%, *P* < 0.0001).

**Table 3:** Unadjusted and Adjusted 12‐Month Complication Rates

	Unadjusted, %			Adjusted, %		
	Aveir VR	Transvenous	*P* value	Aveir VR	Transvenous	*P* value
	(N=2425)	(N=21 335)		(N=2425)	(N=21 335)	
Overall complications	4.2%	5.2%	0.06	4.1%	5.7%	0.02
Embolism and thrombosis						
Thrombosis due to cardiac device	0.2	0.1	0.03	0.2	0.1	0.42
Embolism due to cardiac device	0.09	0.01	0.02	0.09	0.01	0.35
Device‐related complication	2.8	4.6	0.0001	2.8	5.2	0.0002
Device malfunction	1.3	1.6	0.72	1.4	1.7	0.86
Device dislodgement	0.8	1.1	0.21	0.8	1.2	0.21
Infection/inflammatory reaction	0.5	1.1	0.01	0.4	1.4	0.002
Hemorrhage due to cardiac device	0.04	0.5	0.01	0.05	0.5	0.03
Pain due to cardiac device	0	0.2	<0.0001	0	0.2	<0.0001
Stenosis due to cardiac device	0.4	0.4	0.26	0.4	0.5	0.21
Pocket complication	N/A	0.8	…	N/A	…	…
Other complications						
Pericarditis	1.2	0.5	<0.0001	1.1	0.6	0.1
Hemothorax	0	<0.05%	…	0	…	…

### Device Reintervention

Overall device reintervention rates at 12 months, which included device revisions, explants, or replacements, are shown in Figure 2C. Aveir VR LP had 61% lower risk of reinterventions compared with TV‐VVI (adjusted rate 1.2% versus 3.2%; hazard ratio, 0.39 [95% CI, 0.24–0.62]). The most common reason for reintervention was for lead revision in the TV‐VVI group and another LP implant or a replacement in the Aveir VR group. Although the numbers are small, more TV‐VVI pacemakers were replaced with an LP (0.5%) compared with Aveir VRs that underwent a single chamber transvenous implant (0.2%). There were 0% LP revisions compared with 1.4% of TV‐VVI requiring lead revision. The adjusted CRT upgrade rates were low (1.5% Aveir VR versus 1.4% TV‐VVI, *P=*0.77). Other individual device reinterventions are illustrated in Table S7.

### All‐Cause Mortality

There was a difference in long‐term all‐cause mortality between Aveir VR LP and TV‐VVI (Figure 2D). Although the adjusted 30‐day mortality was not significantly different (hazard ratio, 0.89 [95% CI, 0.78–1.01]), the adjusted 12‐month mortality for Aveir VR LP was lower compared with TV‐VVI (17.0% versus 18.2%; hazard ratio, 0.93 [95% CI, 0.87–0.99]) (Table 4).

**Table 4:** All‐Cause Mortality: 30‐Day and 12‐Month

	Aveir VR (N=2425)	Transvenous (N=21 335)	Hazard ratio (95% CI)
30‐d mortality			
Unadjusted	3.5%	3.8%	0.93 (0.75–1.17)
Adjusted	3.5%	4.0%	0.89 (0.78–1.01)
12‐mo mortality			
Unadjusted	17.1%	19.0%	0.89 (0.80–1.00)
Adjusted	17.0%	18.2%	0.93 (0.87–0.99)

## DISCUSSION

This real‐world study comparing transvenous implants and the first commercial Aveir VR implants in US Medicare beneficiaries demonstrates that Aveir VR outcomes were either comparable or better than TV‐VVI pacemakers. Aveir VR had significantly lower device‐related complications at 12 months compared with transvenous pacemakers, both acutely (51% risk reduction) and chronically (47% risk reduction). Aveir VR also had a 61% lower risk of reinterventions compared with transvenous pacemakers at 12 months. Finally, Aveir had a lower 12‐month mortality rate.

The findings of the Aveir VR CED study align with the observations from various registries regarding LP utilization.^4^, ^5^ Patients undergoing LP implant often have more severe comorbidities, such as diabetes, end‐stage renal disease, and dialysis dependence, and are thus at higher risk for bacteremia and subsequent seeding of intravascular hardware. Dialysis patients also require patent vasculature for their hemodialysis treatment. The higher Charlson comorbidity index in the Aveir group compared with the TV‐VVI group was nearly identical to that shown in Micra CED studies.^4^, ^5^ This patient selection likely reflects the advantages of LP technology in reducing pocket and lead‐related complications associated with transvenous pacemakers, such as vascular complications, infections, and fractures.

The overall 30‐day acute complications with Aveir VR were comparable with TV‐VVI, both before and after adjustment. Pneumothorax, a complication that is unique to TV‐VVI pacemakers, occurred in 0.7% of transvenous implants in this study. The first report of the Micra CED study also evaluated 30‐day acute complications and 6‐month complications of Medicare patients implanted with Micra ventricular LP (n=5746) or transvenous VVI pacemakers (n=9662).^4^ While Micra VR had a significantly higher overall acute complication rate compared with TV‐VVI before adjusting for patient characteristics, the study found no significant difference following adjustment (7.7% versus 7.4%; risk difference, 0.3 [95% CI, −0.6 to 1.3]; *P=*0.49).

There was significantly higher pericardial effusion and/or perforation within 30 days among Micra VR compared with TV‐VVI in the Micra CED study (0.8% versus 0.4%; *P=*0.004).^4^ In this current CED study, the cardiac effusion/perforation rates were similar in the Aveir group (0.4%) compared with the TV‐VVI group (0.3%), *P=*0.45. The low rate of pericardial effusions/perforations with early Aveir implants compared with the higher incidence observed with Micra may reflect the design and implant procedural differences between the 2 LP types. Aveir does not require a large amount of forward pressure against the myocardium for deployment and permits passive mapping of sensing and pacing thresholds before deployment. This in turn reduces the amount of recapturing and repositioning required. The soft nitinol protective sleeve at the tip of the delivery catheter also minimizes trauma to the myocardium during LP positioning.^6^ In contrast, the more contemporary Micra AV PAR (Post‐Approval Registry), which is an ongoing prospective single‐arm observational, international registry, there were 11 pericardial effusions among the 801 patients implanted with Micra (1.4%) ‐ 4 requiring surgical intervention, 5 requiring pericardiocentesis, 1 resulting in death, and 1 requiring no intervention.^10^ In the current Aveir CED study, there were 0.4% pericardial effusions/perforations, half of which required intervention.

Importantly, the acute, device‐related complications of particular interest to LP were 51% lower with Aveir VR compared with TV‐VVI, including a significantly lower hemorrhage rate. The Micra CED study also found significantly lower device‐related complications in LPs compared with TV‐VVI.^4^ Device dislodgements were observed in 0.6% of Aveir VR implants compared with 0.8% of TV‐VVI implants with lead dislodgments (*P=*0.55). This may be attributed to the multiple steps that ensure adequate fixation with the Aveir VR implant, including electrical (eg, adequate current of injury, impedance increase during fixation, acute pacing capture threshold after fixation that improves to the passive pacing threshold) as well as mechanical observations (eg, progression of chevron during rotation of LP, tether deflection test after fixation).^6^ Following a year of implant experience post‐FDA approval, the dislodgement rate was 0.4% in the Micra CED^5^ and 0.5% in the Micra AV CED (which started nearly 4 years after Micra VR was approved).^11^

This significant relative risk reduction in device‐related complications (51% at 30 days, 47% at 12 months) is particularly noteworthy because the current Aveir CED study encompasses all Aveir implants done *immediately* after FDA approval to evaluate the early safety profile of this novel helix‐fixation LP, whereas the existing tined‐based LP system does not report early safety results. The first Micra CED study reported on results from implants that started nearly 1 year after FDA approval,^4^ and the Micra AV CED reported implants that started nearly 4 years after the first Micra was FDA approved.^11^ Several studies compared Micra LP to TV‐VVI pacemakers using the National Inpatient Sample (NIS), which only contains inpatient discharge records and therefore a more severe case mix. Alhuarrat et al (2016–2019) found higher in‐hospital mortality for LP implants (2.51% versus 1.28%) and more cardiac complications, including pericardial issues and cardiac arrest.^12^ Vincent et al (2017–2019) also noted higher mortality with LP (5.0% versus 2.4%) but fewer in‐hospital complications (8.0% versus 13.2%).^13^ Yearly data showed decreasing mortality and complication rates from 2017 to 2019. In this study, Aveir VR had a similar 30‐day mortality compared with TV‐VVI.

The 12‐month complication rates were 30% lower with Aveir VR compared with TV‐VVI. Aveir VR had a 47% lower risk of device‐related complications, such as lower risk of infection, compared with TV‐VVI (0.4% versus 1.4%, *P*<0.002). The Micra CED study also reported a lower rate of 6‐month complications compared with TV‐VVI (adjusted hazard ratio, 0.77 [95% CI, 0.62–0.96]; *P=*0.02), and the 3‐year follow‐up also showed a lower rate of chronic complications (HR, 0.68; [0.59–0.78]).^5^ In addition, there were lower infection rates with LPs (<0.2% versus 0.7%, *P*<0.0001) and slightly lower rates of heart failure hospitalization (HR, 0.90; [0.84–0.97]).

The reintervention rates (not including upgrades) were significantly different between Aveir VR and TV‐VVI (1.2% versus 3.2%, *P*<0.0001), which was 61% lower for Aveir at 12 months. Although this current observation period was only 12 months, there was a low incidence in both groups of needing revisions. These results are similar to the 2‐year Micra CED study that showed 38% reduction in reintervention/upgrades with LP compared with TV‐VVI (3.1% versus 4.9%, *P=*0.003) at 2 years.^5^ The Micra 5‐year PAR study reported the rate of system revision at 3 years of 3.2% with Micra compared with 6.6% with TV pacemakers (HR, 0.47 [0.34–0.65], *P*<0.001) (3.2% versus 6.6%).^14^

There was a slight difference in long‐term mortality observed between Aveir VR and TV‐VVI. The Micra CED study reported no difference in 30‐day and 6‐month mortality rates. Aveir VR’s lower long‐term mortality may be clinically significant in the setting of higher comorbidities in the Aveir cohort, where 8.3% of Aveir patients had ESRD compared with 3.7% of TV‐VVI patients and 7.0% of Aveir patients were dialysis‐dependent compared with 2.3% of TV‐VVI patients. In general, patients who are selected for LP implants tend to have more illnesses, such as chronic kidney disease (CKD) and prior infections, a higher Charlson Comorbidity Index (5.0 versus 4.6), and would be expected to have a higher mortality. Among 1748 consecutive patients enrolled in the international, multicenter i‐LEAPER registry of Micra LP implants from June 2015 to October 2023, patients with CKD represented 42.4% of the overall population–33% were CKD stage G3a/G3b, 9.4% were CKD stage G4/G5, and 7% were on hemodialysis.^15^ During a median follow‐up of 39 (interquartile range, 18–59) months, major peri‐procedural complication rates did not differ between groups (normal kidney function=1.8% versus CKD stage‐G3a/G3b=2.9% versus CKD stage‐G4/G5=2.4%, *P=*0.418), but there was a higher all‐cause mortality in CKD stage‐G4/G5 cohort when compared with the normal kidney function group (19.5% versus 9.8%, aHR, 1.9 [95% CI, 1.25–2.89], *P*=0.003).

## LIMITATIONS

This observational study was nonrandomized and relied on administrative claims data, which are not adjudicated by physicians. As a result, there may be some inaccuracies in reporting complications, though the likelihood is low^16^ and would affect both study groups. Statistical methods to address confounding were used, namely, propensity score overlap weights; however, residual confounding may still exist. We included an additional 1.5 years of TV‐VVI implants that preceded the Aveir VR implants in our analysis to increase our sample size of TV‐VVI implants. This temporal disparity may introduce bias attributable to evolving operator experience, health care utilization patterns, or external factors (eg, COVID‐19); however, this bias could go in either direction (eg, higher complications with a newly FDA‐approved device). Another limitation of the CED is the inability to separate LP infections from infections of lines/grafts/fistula and the inability to determine the specific cause of mortality. Finally, the study was based on administrative claims data in a Medicare FFS population, which primarily consists of patients aged ≥65 years, those with disabilities, and individuals with end‐stage renal disease. Consequently, the complication and mortality rates would be expected to be higher than a younger population, potentially overestimating the overall risks of the procedure.

## CONCLUSION

Among patients undergoing Aveir VR implants, the acute and 12‐month device related complication rates as well as total 12‐month complication rates were lower compared with transvenous pacemakers. Long‐term mortality at 12 months was also lower for Aveir VR patients. Additionally, the perforation rates in this study of early Aveir implant experience was low, unlike the higher rates observed in the early and late experience of the Micra LP systems. This real‐world evidence, collected nearly immediately after commercialization, underscores the continued safety and efficacy of the Aveir VR implant for patients with pacing needs.

## Sources of Funding

Abbott provided funding for this study.

## Disclosures

J. Ip ‐ Abbott: consulting fees, steering committee, speaking honoraria; Medtronic: consulting fees; Boston Scientific: data safety monitoring committee, speaking honoraria. P. Brady – None. A. Kroman ‐ Abbott (consultant, speaker), Biotronik (speaker), Philips (speaker, consultant). B. Mondésert ‐consultant/honorarium/protor: Abbott, Boston Scientific, Medtronic, Milestone Pharma, Philips, Cook; research grant: Boston Scientific; speaker: Bayer, Baylis. L. Ganz – Abbott. J. Joseph – Abbott. D. Bettampadi – Abbott. Y. Hu – Abbott. Y. Nabutovsky – Abbott. R. Doshi ‐ clinical research and consulting fees (modest) Abbott.

## Supporting information

Table S1
Table S2
Table S3
Table S4
Table S5
Table S6
Figure S1
