# Supplementary material for: Leadless Versus Transvenous Single‐Chamber Ventricular Pacemakers: Real‐World Evidence From Aveir VR Coverage With Evidence Development Study
Source: J Am Heart Assoc. 2025 Oct 23;14(21):e042471. doi: 10.1161/JAHA.125.042471 (PMC12684545; doi:10.1161/JAHA.125.042471)

SUPPLEMENTAL MATERIAL

Table S1. Leadless VR and TV-VVI device implant codes

CPT and ICD-10 Procedure Codes	
Leadless VR device implant	33274, 02HK3NZ
TV-VVI system implant	33207, 0JH604Z, 0JH605Z, 0JH634Z, 0JH635Z, 0JH804Z, 0JH805Z, 0JH834Z, 0JH835Z

Table S2. Cardiac Implantable Electronic Device (CIED) procedure codes

CPT and ICD-10 Procedure Codes
CIED implant, including leadless single-chamber pacemaker, transvenous single-chamber pacemaker, dual-chamber leadless pacemaker, dual-chamber transvenous pacemaker, implantable cardioverter defibrillator (ICD), or cardiac resynchronization therapy pacemaker (CRT-P) or defibrillator (CRT-D)
"3781","3782","0JH604Z","0JH605Z","0JH634Z","0JH635Z","33207","0JH805Z","0JH835Z","0JH834Z","3783","0JH606Z","0JH636Z","33208","0JH806Z","0JH836Z","0051","0054","0JH609Z","0JH639Z","0JH809Z","0JH839Z","33263","33249","33264","0050","0053","0JH607Z","0JH637Z","0JH807Z","0JH837Z","33214","33229","3780","3783","3787","0JH60PZ","0JH63PZ","0JH80PZ","0JH83PZ","33213","33228","3794","3796","3798","0JH608Z","0JH638Z","0JH808Z","0JH838Z","33221","33230","33240","33262","33270","3779","3781","3782","3785","3786","0JH804Z","33206","33212","33227","0387T","02HK3NZ","02HK0NZ","02HK4NZ","33224","33226","33231","33233","33222","33215","33216","33217","33218","33220","33234","33235","33241","33223","02HK4JZ","02HK3JZ","02HK0JZ","0JPT0PZ","0JPT3PZ","02WA0MZ","02WA3MZ","02WA4MZ","0JWT0PZ","0JWT3PZ","02PA3NZ","02WA3NZ","02H43MZ","02H63KZ","02HK3KZ","02HN0KZ","02HN4KZ","02HK4KZ","02HK0KZ","02H60KZ","02H64KZ","02HL4JZ","02HL3JZ","02HL0JZ","02H44JZ","02H43JZ","02H40JZ","02HL4KZ","02HL3KZ","02HL0KZ","02H44KZ","02H43KZ","02H40KZ","02H73KZ","02H70KZ","02H74KZ","33274","33275","93279","93288","93286","93280","93281","93293","93294","93296","93282","93283","93284","93289","93287","93295","02H63NZ","0JH60PZ","0571T","33225","93743","93744","93731","93734","93741","93640","93641","02HK3DZ","0388T","02PA3DZ","02WA3DZ","0JH805Z","02HK0MZ","02HK3JZ","02HK3MZ","02HK4JZ","02HK4MZ","02HN0JZ","02HN0MZ","02HN3JZ","02HN3MZ","02HN4JZ","02HN4MZ","33238","02PA0MZ","02PA3MZ","02PA4MZ","02PAXMZ","3780","3785","3786","3787","93260","93261","93644","0389T","0390T","0391T","93724","93732","93733","93735","93736","33273","33243","33244","33272","33271","G0448","3771","3772","3770","3773","3776","3775","3777","3789","8945","8946","8947","8948","8949","4B02XSZ","4B02XTZ","3797","02H60JZ","02H63JZ","02H64JZ","02H60MZ","02H63MZ","02H64MZ","02H40MZ","02H44MZ","02HL0MZ","02HL3MZ","02HL4MZ","0052","3795","3796","3794","3796","3798","0JH63FZ","0JH60FZ","0319T","0320T","0321T","0324T","0JPT0PZ","02HN3KZ","02H70JZ","02H73JZ","02H74JZ","02H70MZ","02H73MZ","02H74MZ", “0795T”, “0796T”, “X2H63V9”, “X2HK3V9”, “0823T”

Table S3. Acute complication codes

Acute complications were identified by the presence of one or more of the diagnosis codes listed below (occurring within 30 days of the implant).

Event	Code
ICD-10 Diagnosis Codes	
Acute myocardial infarction during cardiac surgery	(I97.190, I97.790) and (I21.01, I21.02, I21.09, I21.11, I21.19, I21.21, I21.29, I21.3, I21.4, I21.9, I21.A1, I21.A9)
Arteriovenous fistula	I77.0
Cardiac perforation	I97.51
Cardiac tamponade	I31.4
Deep vein thrombosis	I82.401, I82.402, I82.403, I82.409, I82.411, I82.412, I82.413, I82.419, I82.421, I82.422, I82.423, I82.429, I82.4Y1, I82.4Y2, I82.4Y3, I82.4Y9, I82.4Z1, I82.4Z2, I82.4Z3, I82.4Z9, I82.621, I82.622, I82.623, I82.629, I82.A11, I82.A12, I82.A13, I82.A19
Device dislodgement or displacement	T82.12++
Embolism due to cardiac prosthetic devices, implants, and grafts	T82.817+
Hematoma	I97.638
Hemorrhage	I97.618, T82.837+
Hemothorax	(J95.62 or J95.831) plus J94.2, J95.72 plus J94.2
Infection	T82.7+++
Intraoperative cardiac arrest	I97.710 or I97.120
Pain due to cardiac prosthetic device, implant and grafts	T82.847+
Pericardial effusion	I97.51 and (I30.9, I31.3)
Pericarditis	I30.9 or I31.9
Pneumothorax	J95.811 or J95.812
Pulmonary embolism	I26.01, I26.02, I26.09, I26.90, I26.92, I26.99
Stenosis due to cardiac prosthetic device, implant and grafts	T82.857+
Thrombosis due to cardiac prosthetic devices, implants, and grafts	T82.867+

Event	Code
Vascular complication – bleeding or failure of vascular closure device requiring intervention	I97.418 or I97.618
Vascular pseudoaneurysm	I72.4
Other cardiac device malfunction	T82.11++, T82.19++
Pocket complication	T82.897+

Table S4. Chronic complication codes

Chronic complications were identified by the presence of one or more of the diagnosis codes listed below (occurring within 12 months of the implant).

Event	Code
ICD-10 Diagnosis Codes	
Device dislodgement or displacement	T82.12++
Embolism due to cardiac prosthetic devices, implants, and grafts	T82.817+
Hemorrhage	T82.837+
Hemothorax	(J95.62 or J95.831) plus J94.2<br>J95.72 plus J94.2
Infection	T82.7+++
Pain due to cardiac prosthetic device, implant and grafts	T82.847+
Pericarditis	I30.9 or I31.9
Stenosis due to cardiac prosthetic device, implant and grafts	T82.857+
Thrombosis due to cardiac prosthetic devices, implants, and grafts	T82.867+
Other cardiac device malfunction	T82.11++, T82.19++
Pocket complication	T82.897+

Table S5. Device-related reintervention codes

Device-related reinterventions were identified by the presence of a procedure code that indicates a device explant or a single chamber pacemaker (leadless or transvenous) implant WITHOUT the presence of procedure code that indicates an upgrade to a dual-chamber pacemaker, cardiac resynchronization therapy device, or implantable cardioverter defibrillator.

**Event**	**Code**
CPT and ICD-10 Procedure Codes	
Device explant; single-chamber pacemaker implant (leadless or transvenous)	Leadless pacemakers: 33274, 02HK3NZ, 33275, 02PA3NZ, 02WA3NZ, 33207, 0JH604Z, 0JH634Z, 0JH804Z, 0JH834Z, 0JH605Z, 0JH635Z, 0JH805Z, 0JH835Z<br>Transvenous pacemakers: 33207, 0JH604Z, 0JH634Z, 0JH804Z, 0JH834Z, 0JH605Z, 0JH635Z, 0JH805Z, 0JH835Z, 33216, 02HK0JZ, 02HK0MZ, 02HK3JZ, 02HK3MZ, 02HK4JZ, 02HK4MZ, 02HN0JZ, 02HN0MZ, 02HN3JZ, 02HN3MZ, 02HN4JZ, 02HN4MZ, 33215, 33218, 02WA0MZ, 02WA3MZ, 02WA4MZ, 33234, 33238, 02PA0MZ, 02PA3MZ, 02PA4MZ, 02PAXMZ, 33227, 0JWT0PZ, 0JWT3PZ, 33233, 0JPT0PZ, 0JPT3PZ, 33222, 33274, 02HK3NZ
Upgrade	33221, 33224, 33225, 33229, 33230, 33231, 33240, 33249, 33262, 33263, 33264, 33270, 33206, 33208, 33213, 33214, 33228, 0571T, 0JH607Z, 0JH609Z, 0JH637Z, 0JH639Z, 0JH807Z, 0JH809Z, 0JH837Z, 0JH839Z, 0JH608Z, 0JH638Z, 0JH808Z, 0JH838Z, 0JH60PZ, 0JH60PZ, 0JH63PZ, 0JH80PZ, 0JH83PZ, 0JH606Z, 0JH636Z, 0JH806Z, 0JH836Z, 02H60JZ, 02H63JZ, 02H64JZ, 02H43JZ, 02H40JZ, 0795T, 0796T, 0797T, 0801T, 0802T, 0803T, X2H63V9, X2HK3V9

Table S6. Clinical characteristics codes

Clinical Characteristics	Code
Atrial and Ventricular Arrhythmias	
Atrial Fibrillation	Any codes in the following ranges: I48.0-I48.2 or I48.91
Atrial Flutter	Any codes in the following ranges: I48.3-I48.4 or I48.92
History of Supraventricular Tachycardia	I47.1
History of Ventricular Arrythmia	Any of the following codes: Z86.74 or I47.0 or I47.2 or I49.01 or I49.02 or I49.3
	• Myocardial infarction: I21.x, I22.x, I25.2<br>• Congestive heart failure: I09.9, I11.0, I13.0, I13.2, I25.5, I42.0, I42.5 - I42.9, I43.x, I50.x, P29.0<br>• Peripheral vascular disease: I70.x, I71.x, I73.1, I73.8, I73.9, I77.1, I79.0, I79.2, K55.1, K55.8, K55.9, Z95.8, Z95.9<br>• Cerebrovascular disease: G45.x, G46.x, H34.0, I60.x - I69.x<br>• Dementia: F00.x - F03.x, F05.1, G30.x, G31.1<br>• Chronic pulmonary disease: I27.8, I27.9, J40.x - J47.x, J60.x - J67.x, J68.4, J70.1, J70.3<br>• Rheumatic disease: M05.x, M06.x, M31.5, M32.x - M34.x, M35.1, M35.3, M36.0<br>• Peptic ulcer disease: K25.x - K28.x<br>• Mild liver disease: B18.x, K70.0 - K70.3, K70.9, K71.3 - K71.5, K71.7, K73.x, K74.x, K76.0, K76.2 - K76.4, K76.8, K76.9, Z94.4<br>• Diabetes without chronic complication: E10.0, E10.1, E10.6, E10.8, E10.9, E11.0, E11.1, E11.6, E11.8, E11.9, E12.0, E12.1, E12.6, E12.8, E12.9, E13.0, E13.1, E13.6, E13.8, E13.9, E14.0, E14.1, E14.6, E14.8, E14.9<br>• Diabetes with chronic complication: E10.2 - E10.5, E10.7, E11.2 - E11.5, E11.7, E12.2 - E12.5, E12.7, E13.2 - E13.5, E13.7, E14.2 - E14.5, E14.7<br>• Hemiplegia or paraplegia: G04.1, G11.4, G80.1, G80.2, G81.x, G82.x, G83.0 - G83.4, G83.9<br>• Renal disease: I12.0, I13.1, N03.2 - N03.7, N05.2 - N05.7, N18.x, N19.x, N25.0, Z49.0 - Z49.2, Z94.0, Z99.2<br>• Any malignancy, including lymphoma and leukemia, except malignant neoplasm of skin: C00.x -C26.x, C30.x - C34.x, C37.x - C41.x, C43.x, C45.x - C58.x, C60.x - C76.x, C81.x - C85.x, C88.x, C90.x - C97.x

Clinical Characteristics	Code
	• Moderate or severe liver disease: I85.0, I85.9, I86.4, I98.2, K70.4, K71.1, K72.1, K72.9, K76.5, K76.6, K76.7<br>• Metastatic solid tumor: C77.x - C80.x<br>• AIDS/HIV: B20.x - B22.x, B24.x
Chronic Obstructive Pulmonary Disease	Any code in the following ranges: J43.0-J43.9 or J44.0-J44.9 or J47.0-J47.9 or J60-J63.6
Coronary Artery Disease	Any code in the following ranges: I25.10-I25.119 or I25.700-I25.739 or I25.790-I25.799 or I25.810 or I25.750-I75.769 or I25.811-I25.812 or I25.82-I25.84
Diabetes	Any code in the following ranges: E08.00-E08.9 or E09.00-E09.9 or E10.10-E10.9 or E11.00-E11.9 or E13.00-E13.9)
Heart Failure	Any code in the following ranges: I09.81 or I11.0 or I13.0 or I13.2 or I50.20-I50.9 or I97.130-I97.131
Hyperlipidemia	E78.1-E78.5
Hypertension	Any code in the following ranges: I10 or I11.0-I11.9 or I12.0-I12.9 or I13.0-I13.2 or I15.0-I15.9 or I16.0-I16.9 or I97.3
Peripheral Vascular Disease	Any code in the following ranges: I70.201-I70.299 or I70.301-I70.799 or I73.00-I73.9
Prior Cardiovascular Events And Procedures	
Prior Coronary Artery Bypass Graft	Any code in the following ranges: Z95.1 or T82.211A-T82.218S or I25.700-I25.739 or I25.790-I25.799 or I25.810
Prior Acute Myocardial Infarction	Any code in the following ranges: I25.2 or I21.01-I21.4
Prior Percutaneous Coronary Intervention	Any code in the following ranges: Z95.5 or Z98.61 or T82.855A-T82.855S
Concomitant Atrial Ablation	93650 or 93653 or 93656 or 93657 or 02583ZZ + (I48.0-I48.2 or I48.91)
Concomitant Transcatheter Aortic Valve Replacement	33361or 33362 or 33363 or 33364 or 33365 or 33366 or 02RF38Z or 02RF38H
Prior Transcatheter Aortic Valve Replacement	33361or 33362 or 33363 or 33364 or 33365 or 33366 or 02RF38Z or 02RF38H
Renal Disease	Any code in the following ranges: K76.7 or N17.0-N17.9 or N18.1-N18.9 or N19 or N28.9 or N99.0 or R39.2
End Stage Kidney Disease	N18.5, N18.6, I12.0, I13.11
Dialysis Dependence	Z49.01, Z49.02, Z49.31, Z49.32, Z91.15, Z99.2,

Clinical Characteristics	Code
	'031509D', '031509F', '031509V', '03150AD', '03150AF', '03150AV', '03150JD', '03150JF', '03150JV', '03150KD', '03150KF', '03150KV', '03150ZD', '03150ZF', '03150ZV', '031609D', '031609F', '031609V', '03160AD', '03160AF', '03160AV', '03160JD', '03160JF', '03160JV', '03160KD', '03160KF', '03160KV', '03160ZD', '03160ZF', '03160ZV', '031709D', '031709F', '031709V', '03170AD', '03170AF', '03170AV', '03170JD', '03170JF', '03170JV', '03170KD', '03170KF', '03170KV', '03170ZD', '03170ZF', '03170ZV', '031809D', '031809F', '031809V', '03180AD', '03180AF', '03180AV', '03180JD', '03180JF', '03180JV', '03180KD', '03180KF', '03180KV', '03180ZD', '03180ZF', '03180ZV', '031909F', '03190AF', '03190JF', '03190KF', '03190ZF', '031A09F', '031A0AF', '031A0JF', '031A0KF', '031A0ZF', '031B09F', '031B0AF', '031B0JF', '031B0KF', '031B0ZF', '031C09F', '031C0AF', '031C0JF', '031C0KF', '031C0ZF', '041C09D', '041C09F', '041C0AD', '041C0AF', '041C0JD', '041C0JF', '041C0KD', '041C0KF', '041C0ZD', '041C0ZF', '041K09S', '041K0AS', '041K0JS', '041K0KS', '041K0ZS', '041L09S', '041L0AS', '041L0JS', '041L0KS', '041L0ZS'
Tricuspid Valve Disease	Any code in the following ranges: I07.I-I07.9 or I08.1-I08.3 or I36.0-I36.9 or Q22.4 or Q22.8-Q22.9
COVID-19	U07.1, B97.29

Table S7. Rates of Individual Reinterventions

Aveir VR (N = 2,425)			*Transvenous (N = 21,335)*	
	N (%)*			N (%)
Leadless implant or replacement	17 (0.7)		Replacement with Leadless	78 (0.4)
Leadless removal	<11		Device replacement	110 (0.5)
Leadless revision	<11		Device removal	104 (0.5)
Single chamber transvenous implant	<11		Device revision	43 (0.2)
			Lead-related reintervention	248 (1.2)

*To comply with the Centers for Medicare and Medicaid Services (CMS) cell size suppression policy, a cell containing a value of 1 to 10 cannot be reported directly; therefore, '< 11' is used to display a value of 1 to 10.

Individual device reintervention rates were not compared between treatment groups and therefore are not adjusted.

Figure S1. Propensity Score Overlap Weight Output

Standardized Difference Love Plot

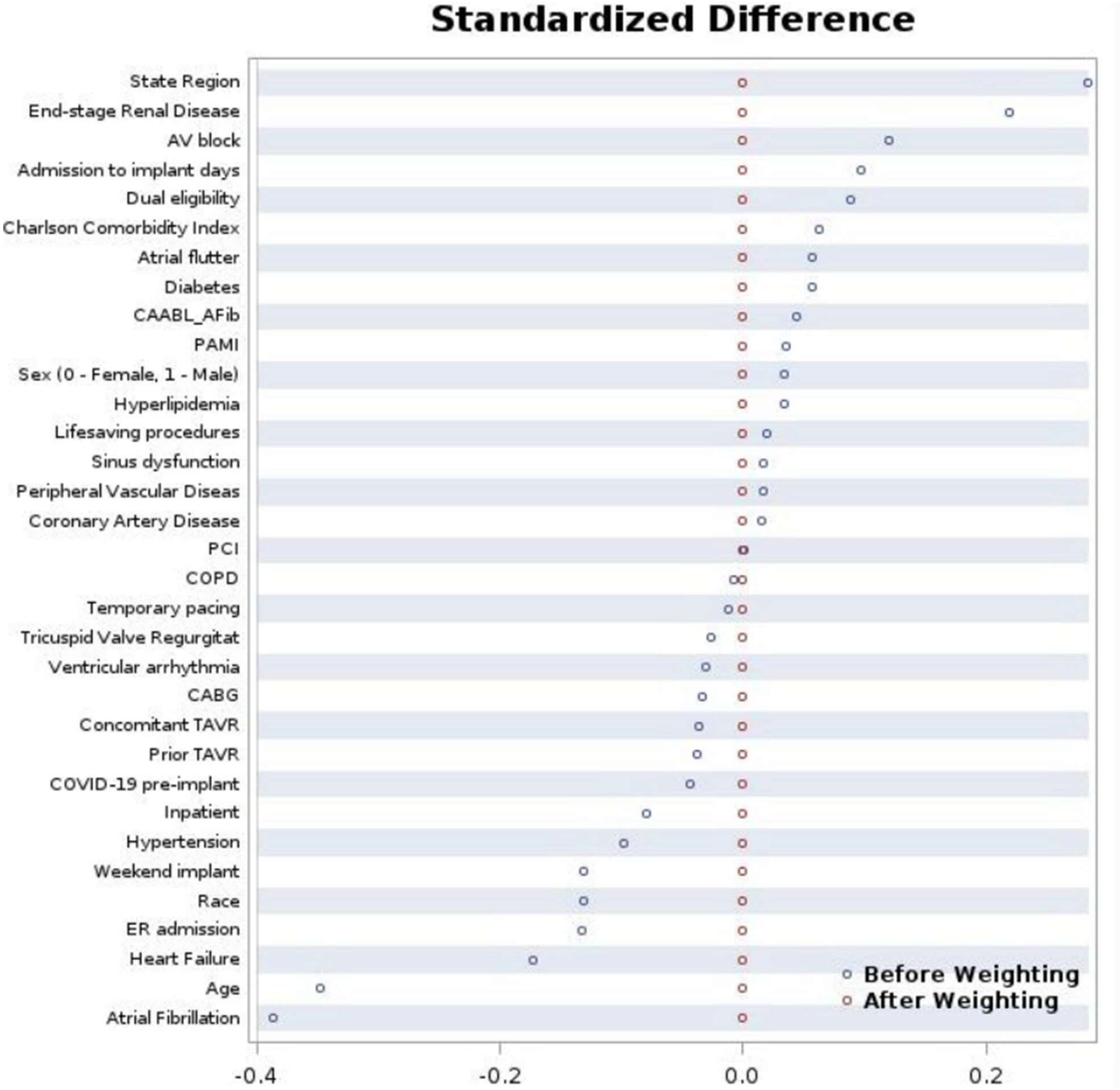

Propensity Score Distributions: Overlap between Aveir VR and Transvenous

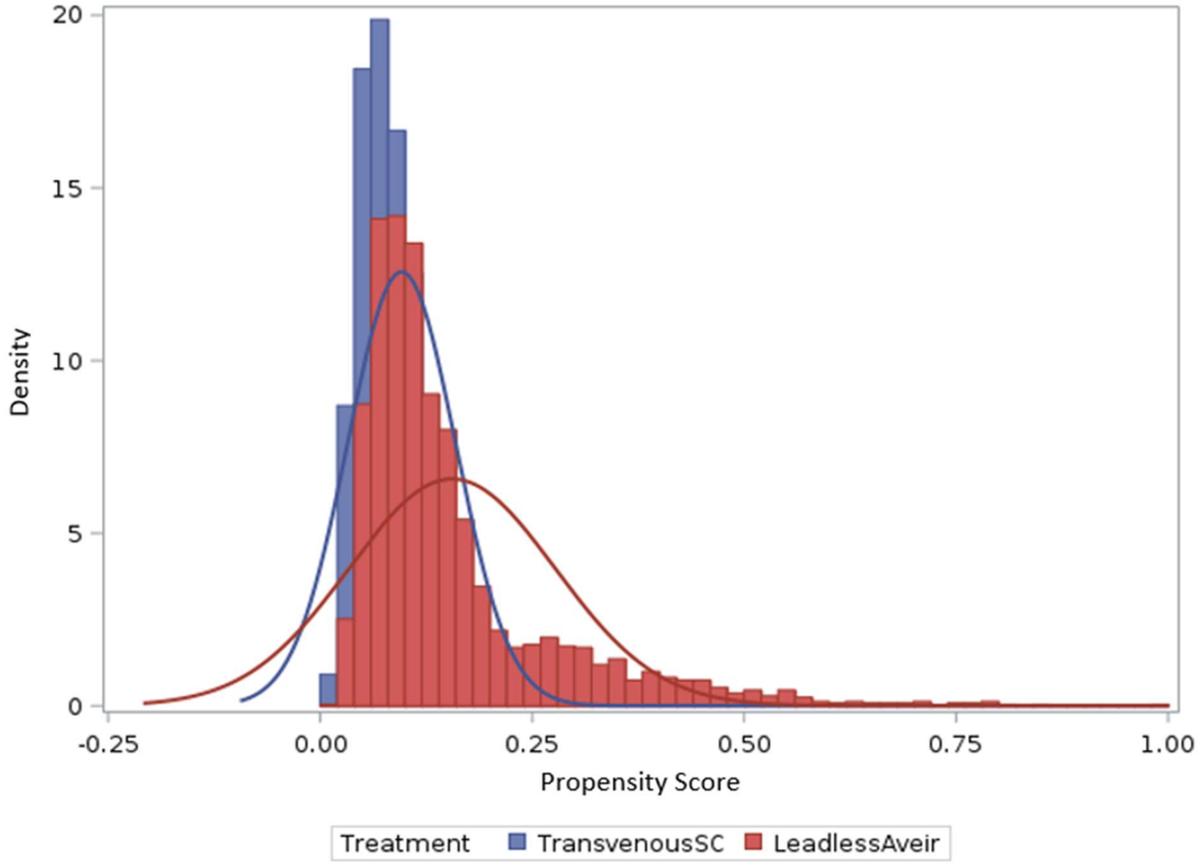

Supplement: Supplementary file 1 — Table S1 Table S2 Table S3 Table S4 Table S5 Table S6 Figure S1 [file JAH3-14-e042471-s001.pdf]
